# Impact of Family Caregiver Training on Hospitalization During Medicare Home Health Care

**DOI:** 10.1093/geroni/igaa057.812

**Published:** 2020-12-16

**Authors:** Julia Burgdorf, Jennifer Wolff

**Affiliations:** Johns Hopkins Bloomberg School of Public Health, Baltimore, Maryland, United States

## Abstract

Medicare home health providers are required to offer family caregiver training; however, there is little information regarding the impact of family caregiver training on patient outcomes in home health or other care delivery settings. A better understanding of this relationship is necessary to guide development of caregiver training interventions and inform policy discussions surrounding family caregiver training access. This research assesses whether and how unmet need for family caregiver training is associated with acute care utilization during Medicare home health. We examine 1,217 (weighted n=5,870,905) fee-for-service Medicare beneficiaries who participated in the National Health and Aging Trends Study (NHATS) and received Medicare-funded home health care between 2011-2016. We link NHATS data with home health patient assessments and Medicare claims, drawing measures of family caregivers’ need for training from home health clinician reports and determining provision of training from Medicare claims. Using weighted, multivariable logistic regressions, we model the marginal change in probability of acute care utilization during home health as a function of family caregivers’ unmet need for training. We found that older adults whose family caregivers had an unmet need for training had a probability of acute care utilization during home health that was 18 percentage points (p=0.001) greater than those whose family caregivers both needed and received training, holding all covariates at their means. Findings support the importance of connecting family caregivers to training resources and suggest one avenue by which investing in caregiver training may be cost-effective for integrated payers and providers.

